# Better Understanding the Potential Importance of Florida Roadside Breeding Habitat for the Monarch

**DOI:** 10.3390/insects9040137

**Published:** 2018-10-11

**Authors:** Jaret Daniels, Chase Kimmel, Simon McClung, Samm Epstein, Jonathan Bremer, Kristin Rossetti

**Affiliations:** 1 McGuire Center for Lepidoptera and Biodiversity, Florida Museum of Natural History, 3215 Hull Road, Gainesville, FL 32611, USA; cbkimmel@ufl.edu (C.K.); simon.mcclung@gmail.com (S.M.); sepstein@flmnh.ufl.edu (S.E.); jbremer@ufl.edu (J.B.); khyla@ufl.edu (K.R.); 2 Department of Entomology and Nematology, University of Florida, 1881 Natural Area Drive, Steinmetz Hall, Gainesville, FL 32611, USA

**Keywords:** monarch butterfly, migration, milkweed, roadside habitat, conservation, vegetation management, mowing

## Abstract

The North American monarch butterfly (*Danaus plexippus*) population has declined significantly over the past two decades. Among the many other factors, loss of breeding habitat has been implicated as a potential leading driver. In response, wildlife agencies and conservation practitioners have made a strong push to restore and conserve milkweeds on both wild and managed landscapes including agricultural lands as well as transportation and utility rights-of-way. Roadsides in particular have been emphasized as a targeted landscape for monarch habitat restoration. While much attention has been focused on habitat in California, along the I-35 corridor from Texas to Minnesota, and more broadly across the agricultural Midwest, research on the occurrence of roadside breeding habitat and the development of best vegetative management practices conducted in the Deep South has been limited. We sampled roadside verges in north-central Florida for the presence of two early season milkweed species, that are particularly important for early season monarch recolonization, *Asclepias tuberosa* and *Asclepias humistrata*. Our findings suggest that roadsides harbor extensive populations of the target milkweeds with the vast majority of plants occurring on the back slope of the verge. Alterations to current roadside mowing frequency and scope are needed to effectively conserve these populations and ensure that they are available for use by the monarch.

## 1. Introduction

The continental and multigenerational annual migration of the iconic monarch butterfly (*Danaus plexippus*) is considered one of the most spectacular natural phenomena on the planet. Well documented declines of the eastern population show significant losses during the past two decades, severely threatening the persistence of the migratory life cycle [1,2]. In north-central Florida, the abundance of adult and immature monarchs declined by around 80% since 2005 [3]. While the drivers affecting the population dynamics and declines are likely complex, recent studies have shown strong correlations with the loss of milkweed (*Asclepias* sp.) host plants across their breeding range [4]. Thus, priority conservation efforts have focused on the creation, restoration, and maintenance of breeding habitat in order to reach the strategic goal set by the White House of increasing the eastern population of the monarch to 225 million butterflies occupying an area of approximately 6 hectares in the overwintering grounds in Mexico by 2020 [5,6]. Despite the tremendous attention and funding commitments generated to help recover and improve breeding habitat, the majority of the efforts have been focused in California, along the I-35 corridor from Texas to Minnesota, and throughout the agricultural Midwest. Although these regions likely contribute significantly to the overwintering populations, expanding the geographic focus to also include locations in the Southeast is needed if effective long-term monarch recovery is to be achieved.

Considerable research, including extensive cardenolide fingerprinting data, has supported the hypothesis that the early spring monarchs collected from Texas to northern Florida (e.g., Gulf Coast/Deep South) represent individuals that are returning from overwintering sites in Mexico and thus represent an important component of the annual stepwise northward migration to recolonize eastern North America [7,8,9,10]. Trans-national population models suggest that restoring habitats in southern and north-central parts of the monarchs’ range give the highest projected growth rates [11]. As a result, efforts supporting adequate and high quality breeding habitat in this region are important to help facilitate recolonization.

Of the 18 milkweed species that occur in Southeast United States Coastal Plain, pinewoods milkweed (*Asclepias humistata*) is arguably one of the most important to spring monarch breeding as it begins vegetative growth from late February through March, just as adult monarchs begin returning to the region each spring. It remains vegetative through early summer thereby supporting more than one monarch generation annually. Found in a variety of sandy, well-drained habitats from pinelands to pastures, it is frequently encountered along roadsides—landscapes not typically impacted by prescribed fire, cattle, or development but highly beneficial for monarch breeding. Butterflyweed (*Asclepias tuberosa*) is another early-season milkweed that often co-occurs with *A. humistrata* in many of the same habitats and is frequently utilized by the monarch as a larval host in Florida.

The conservation and expansion of available breeding habitat on non-traditional managed landscapes such as road verges (managed vegetative area located immediately beyond the road shoulder) and other rights-of-way is identified as a primary goal in the Pollinator Research Action Plan [5] to help provide substantial, continuous habitat for monarchs and other insect pollinators. High-density milkweed populations on such landscapes may also offer opportunities for the collection of needed quantities of local or regional ecotype seed not currently available for larger scale restoration or revegetation projects.

In Florida, the Department of Transportation (FDOT) is responsible for the management and care of over 75,000 hectares or ½ of one percent of the entire land area in the state [12]. Of this managed land along the State Highway System, approximately half is thought to be vegetated. These roadside areas represent likely reservoirs of larval host resources and a wide diversity of blooming plants for adult forage. Despite this potential, limited data is available on the utility of Florida roadsides to support key milkweed populations for monarch breeding. Our goal was to survey FDOT-maintained roadsides in north-central Florida for key milkweed populations important for spring monarch recolonization, primarily *A. humistrata* and *A. tuberosa*, and identify high density locations that may be particularly important to conserve.

## 2. Materials and Methods

### 2.1. Study Area and Road Prioritization

**Figure 1:**
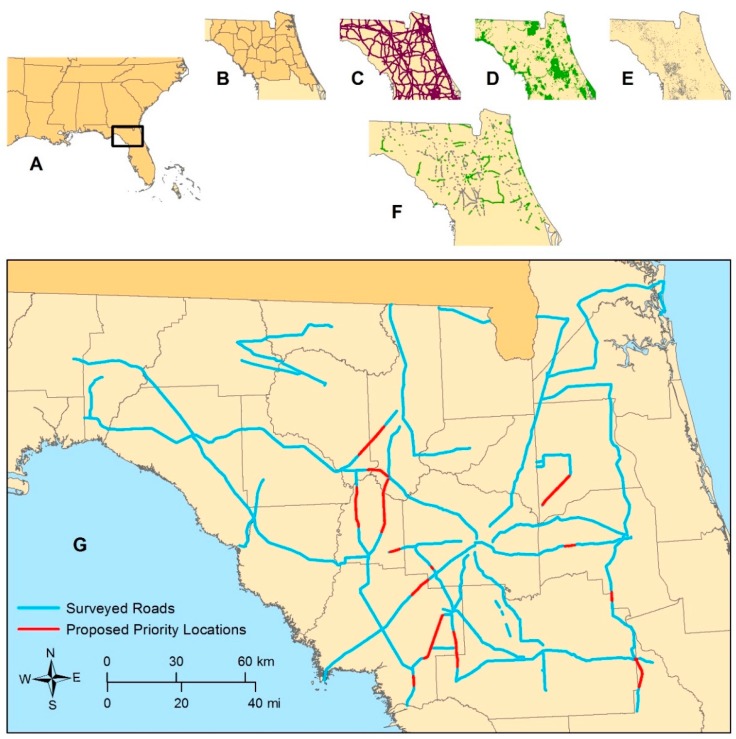
Maps showing project location (**A**), initial survey selection process (**B**–**E**) and resulting output (**F**), and roads surveyed as well as proposed priority areas (**G**). The selection process to determine higher priorities areas (**F**) was performed by selecting counties in north central Florida (**B**) along state roads (**C**) that were beside conservation lands (**D**) or agricultural areas (**E**). All proposed priority location delineations include a 500 m buffer on either side to more effectively enable Florida Department of Transportation to evaluate a modified mowing scheme.

The target geographic scope included a broad 22 county area in north-central Florida that directly overlapped with the known range of *A. humistrata*. The specific priority counties were Alachua, Baker, Bradford, Clay, Columbia, Dixie, Duval, Flagler, Gilchrist, Hamilton, Jefferson, Lafayette, Levy, Madison, Marion, Nassau, Putnam, St. Johns, Suwannee, Taylor, Union, and Volusia. ArcMap 10.2.2 (ERSI, Redlands, CA, USA) [13] was used to initially prioritize roads and roadway sections for survey based on adjacent land cover (i.e., conservation and agricultural lands). Conservation land layers were downloaded from the Florida Natural Areas Inventory (FNAI) [14] and agricultural lands from Florida Fish and Wildlife Conservation Commission [15]. Agricultural types included: Agriculture, Citrus, Cropland/Pasture, Field Crops, Floriculture, Fruit Orchards, Orchards/Groves, Other Agriculture, Row Crops, and Specialty Farms. A 30 m buffer was added to the land cover types selected to ensure that they would overlap roadways during analysis. All state managed roadways [16] were then clipped from the aforementioned statewide layers. Next, the intersection of the roadway layer containing the land cover type polygons was identified. The resulting roadway sections were subsequently transcribed onto maps and used as priority survey locations. In most situations however, the entire road distance was comprehensively surveyed (Figure 1).

### 2.2. Roadside Surveys

All surveys were conducted between 28 March and 29 June 2016. This time period corresponds to the initial date when *A. humistrata* plants begin vegetative growth and are thus reliably visible in the landscape but before many plants begin to senesce. Target roadsides were initially surveyed visually from a moving vehicle. At least two surveyors were present in a vehicle at all times for safety and in order to effectively scan both sides of the road. Once appropriate target habitat or roadside conditions were identified (i.e., well-drained, sandy soil; associated plants such as *Quercus laevis*; steep back road verge slope with exposed sandy soil), travel speed was reduced. If any target milkweed species was identified, the research personnel would stop the vehicle by pulling safely off the side of the road and survey the specific location in greater detail on foot. Using a Keson^®^ Steel professional digital measuring wheel, the distance from the paved road edge to the beginning of the nearest plant or plants in meters was recorded along with the distance parallel to the road between the first and last plant. This provided the distance to road and overall approximate length of any located milkweed populations, including individual plants (i.e., singletons).

For each site surveyed, a detailed data sheet was completed. In addition to the measurements described above, information recorded included the names of the researchers conducting the survey, date, total number of plants, development stage of plants (e.g., immature flower buds, flowering, mature seed pods, post-pod with seeds released), leaf color (e.g., green, purple, mixed), presence of monarch eggs or immature stages, nearest intersection or approximate descriptive location on road, and any specific notes of interest. While every patch of milkweed was surveyed, each plant within the patch was not meticulously inspected for the presence of eggs and larvae. However, if eggs and larvae were observed it was recorded. A GPS waypoint was also taken for each location. If the site had multiple plants, a waypoint between the two farthest plants or several points spread across the area covered were taken and recorded.

Following data collection, the survey date, surveyors’ initials, and GPS waypoint were written on a small whiteboard. The whiteboard was then placed on the ground in the site and two photographs were taken: One with a close-up photo(s) of plant(s) with a ruler for scale, and one showing the plants’ location in relation to the road. Each photo included the whiteboard. At the end of each field day, all site photographs were downloaded, and all data was entered on a comprehensive master geo-referenced Microsoft Excel spreadsheet.

## 3. Results

**Figure 2:**
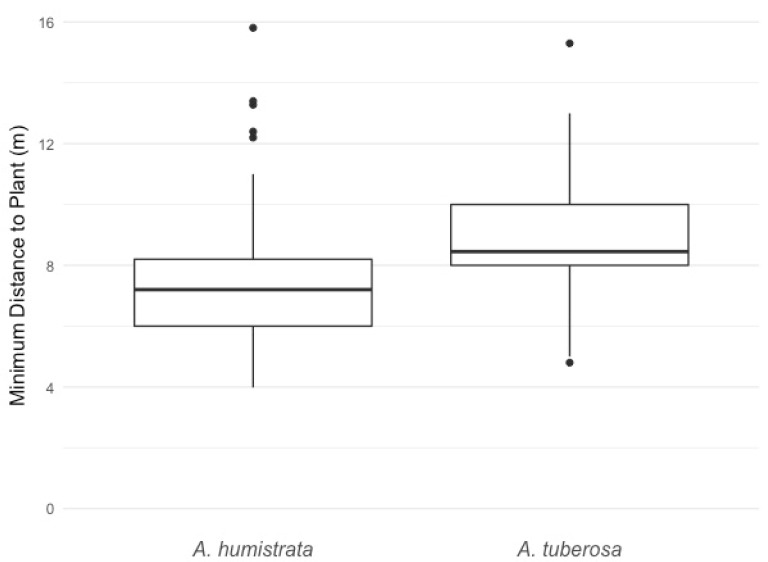
Box plot representing occurrences of the minimum distance between the road edge and plant among the two different milkweed species, *A. humistrata* (*n* = 169) and *A. tuberosa* (*n* = 29).

**Figure 3:**
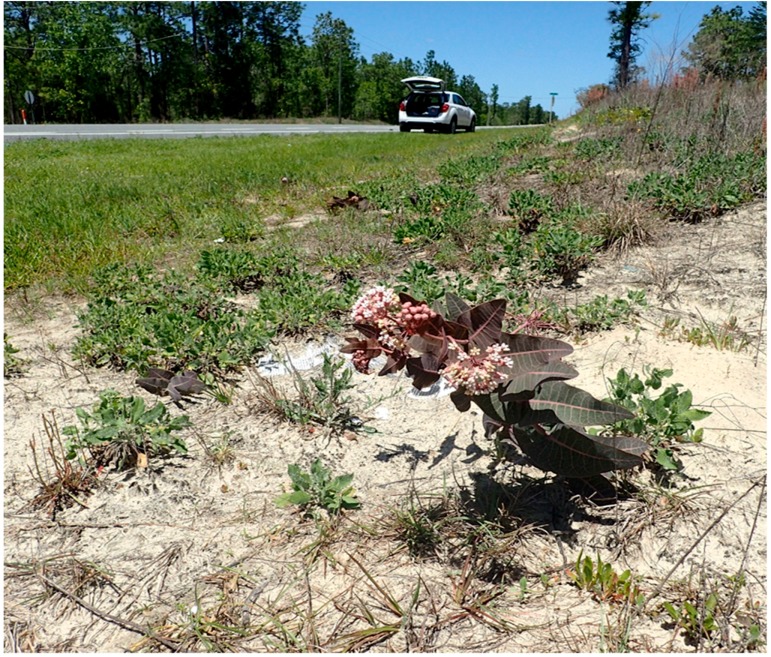
Road verge showing a population of pinewoods milkweed (*Asclepias humistrata*) with plants growing on the back upward sloped portion where exposed sand occurs.

Over 1950 km of FDOT-maintained roadsides were comprehensively surveyed for the target milkweed species. In total, 169 distinct *A. humistrata* and 29 *A. tuberosa* locations were identified across the 22 county area. This included both individual plants (singletons) and populations (multiple plants occurring at a single location). For all locations, plants in the verge occurred well away from the edge of the paved road (Figure 2). In most cases, this resulted in the majority of individual plants occurring on the back upward sloped portion of the verge, often in areas with exposed sandy soils and reduced vegetation (Figure 3). The plants encountered represented a continuum of new recruits to older, well-established plants. A large portion of the populations encountered were older, productive plants with blooms, pods, and seed set confirmed. Of the 169 locations surveyed, 18 (or 9.1%) of them were observed to have monarch eggs or larvae present.

Based on the survey data, plant densities ranged from 1 to (an estimated) 500 individuals per site. As a result, plant locations were subsequently categorized as either high or low density. This was done for the purposes of identifying proposed priority locations for evaluating current and future vegetation management (e.g., mowing, selective herbicide application). Sites were considered high density if they contained at least 1 plant for every 2 m of road verge and were at least 10 m in overall length (parallel to the road) from the first plant to the last plant. Alternatively, sites were considered low density if they failed to meet one or both of these requirements. Based on these criteria, a total of 35 *A. humistrata* and 0 *A. tuberosa* locations were characterized as high density, proposed priority locations (Figure 1). As *A. tuberosa* is more likely to occur singly and is much more difficult to immediately identify in the landscape from a distance unless in flower, the lower numbers recorded may be an artifact related to detectability.

## 4. Discussion

Our study represents one of the first such efforts to comprehensively assess and map monarch habitat in Florida. It indicates that FDOT-maintained roadsides harbor a considerable number of milkweed populations important to and actively utilized by the monarch butterfly during the spring recolonization of the state, especially when other habitat is scarce. While we found that 9% of the patches containing milkweed had monarch eggs or larvae, this number is expected to be higher as not every plant within all patches was thoroughly inspected. In many cases, milkweed populations were characterized as high density and comprised of older, reproductive plants. The potential conservation benefits of roadside habitat for the monarch has been widely suggested, particularly if such landscapes are properly managed [17,18,19,20]. Maintaining roadside vegetation for road integrity, safety, and aesthetics represents a significant cost to FDOT, with the greatest proportion of this expenditure going to mowing. As Florida has a humid subtropical climate, vegetation growth continues in most regions throughout the year. Correspondingly, roadside mowing cycles vary widely across FDOT districts and increase from north to south in the state, ranging from 6 to 24 annually based on location [12]. The number, timing and extent (area of road verge) of mowing cycles is likely to impact the availability of milkweed resources (vegetative and floral) to the monarch and other insect pollinators, as well as successful plant reproduction, seed dispersal, and population persistence and expansion. Increased mowing cycles or improper timing of mowing may regularly trim/damage plants, reduce or prevent plant flowering and reproduction, hinder or prevent recruitment, and cause direct mortality to immature stages of the monarch. The extent of the road verge that is regularly mowed has equally significant consequences.

Our findings, indicating that the majority of milkweed populations occurred well away from the paved road and often on the back, steeply sloped section of the road verge, have direct conservation and management implications. Specifically, identified milkweed populations would suffer little or no impact from mowing if FDOT targeted only the safety strip (the 3–4.5 m relatively flat unobstructed rights-of-way section directly adjacent to the inside and outside lanes of pavement) instead of the entire clear zone. In some locations where mowing was reduced, observed plants had no vegetative damage and were able to successfully flower and set seed. Norcini [17] additionally showed that there were no adverse impacts for roadside integrity (e.g., erosion, turf quality) or safety over time from such a modified vegetation management regime, and that overall roadside aesthetics improved particularly in spring due to a resulting increase in the occurrence and density of native herbaceous flowering plants. Moreover, Harrison [12] concluded that by implementing sustainable practices such as reduced mowing, FDOT would realize a 30% annual saving in vegetation management costs and more than double the total value (estimated at more than $1 billion U.S.) of the ecological services such as carbon sequestration, runoff and erosion control, insect pollination and natural pest control, and invasive species resistance provided by roadside vegetation.

In many cases, it appears that roadside vegetation management may often benefit milkweed populations. While *A. humistrata* is found primarily in sandhill and scrub habitats throughout Florida, we observed it to be absent or nearly so on numerous occasions from undeveloped or conservation lands directly adjacent to surveyed roadsides presumably due to fire suppression. In contrast, mowing mimics many of the effects of fire and thus likely aids in the maintenance and persistence of milkweed populations on roadsides. Nonetheless, to maximize vegetative growth, flowering, follicle production, and seed dispersal of the target milkweed species as well as potential use by monarch butterflies and other insect pollinators, mowing of the entire clear zone (which includes the back upward slope of the verge) should be avoided from February through July in most years. Fortuitously in many areas, including a number of those that harbor identified high density milkweed populations, FDOT already implements special management practices (e.g., reduced mowing) to increase the abundance and visibility of natural or augmented wildflower populations, especially during the spring season when prolific roadside flowering typically occurs. Productive high density milkweed populations may also be useful as a source of local ecotype seed for restoration.

The overall utility of roadside habitat for monarch conservation is still much debated. The use of such managed landscapes demands attention to a number of somewhat unique considerations especially when compared to other systems. While some studies have acknowledged that roadsides have potential to benefit monarch butterfly, or more broadly, insect pollinator conservation [18,19,20,21,22], others have demonstrated potential pitfalls that may deleteriously affect the monarch or otherwise suggest such landscapes are suboptimal [21,22,23,24,25,26,27]. The lack of comprehensive research in this area in terms of the number of studies and overall geographic scale and focus, severely limit interpretation. In addition, milkweed species diversity and habitat affinities, roadside management practices, and potential anthropogenic threats such as increased sodium from salt application vary considerably across the U.S. As a result, conservation recommendations should be made cautiously and within context. It is clear that considerably more research needs to be conducted in order to more thoroughly document the presence of milkweeds and monarch breeding along roadsides as well as the myriad of potential benefits and hazards such landscapes may present.

## 5. Conclusions

This study provides greater detail of the roadside distribution of two milkweed species important for the spring recolonization of Florida by the monarch. It shows that roadsides harbor extensive milkweed populations with the vast majority of plants occurring on the back slope of the verge. Modifications to roadside mowing timing, frequency and scope are needed to effectively conserve these populations and ensure that they are available for use by the monarch and other insect pollinators.
